# Neutrophil-to-lymphocyte, platelet-to-lymphocyte and lymphocyte-to-monocyte ratios, any association with metabolic syndrome? 

**DOI:** 10.22088/cjim.14.3.567

**Published:** 2023

**Authors:** Versa Omrani-Nava, Mahmood Moosazadeh, Adeleh Bahar, Akbar Hedayatizadeh-Omran, Abdolrahim Ahmadi, Reza Alizadeh-Navaei

**Affiliations:** 1 Gastrointestinal Cancer Research Center, Non-communicable Diseases Institute, Mazandaran University of Medical Sciences, Sari, Iran; 2 Diabetes Research Center, Mazandaran University of Medical Sciences, Sari, Iran

**Keywords:** Metabolic syndrome, neutrophil-to-lymphocyte ratio (NLR), platelet to lymphocyte ratio (PLR), lymphocyte-to-monocyte ratio (LMR)

## Abstract

**Background:**

Metabolic syndrome is a critical health concern associated with an elevated risk of chronic health problems including cardiovascular disease and diabetes. There are shreds of evidence that novel inflammatory ratios including neutrophil-to-lymphocyte, platelet-to-lymphocyte and lymphocyte-to-monocyte ratios serve as prognostic biomarkers for metabolic syndrome (MetS). This hypothesis was investigated in a cohort of the Iranian population.

**Methods:**

selection of MetS + subjects was based on the National Cholesterol Education Program Adult Treatment Panel III criteria 3 (NCEP ATP 3). The control group consisted of participants negative for any of the five MetS criteria. Demographic and laboratory data were extracted from the Tabari cohort study.

**Results:**

A total of 1930 subjects including 965 Mets positive and 965 MetS criteria negative participants were evaluated. Diabetes (84.8%), hypertension (48.9%), hypertriglyceridemia (81.7%), low HDL cholesterol (70.3%), and high waist circumference (78.9%) were observed in patients. There were no differences between NLR (1.66±0.71 vs. 1.69±0.72 P=0.42), LMR (11.23±3.13 vs. 11.30±11.99, P= 0.86) and PLR (113.85±68.67 vs 114.11±35.85, P=0.91) between case and control groups, respectively. Logistic regression analysis revealed no association between ratios and MetS risk even after adjusting for potential confounders including age, gender, living place, and BMI.

**Conclusion:**

In a relatively large population from Northern Iran, no association was observed between CBC-derived inflammatory ratios and the presence of MetS.

Metabolic syndrome (MetS) with a global prevalence of about 25%, is recognized as a common disease worldwide and an important health concern (1). Hyperglycemia and insulin resistance, abdominal obesity, dyslipidemia and hypertension which are important criteria for chronic health issues such as cardiovascular disease, diabetes and cancer are components of MetS (2,3). High rate of MetS in developing countries including Iran needs special attention of health policy makers because of impaired quality of life affected by it (4). There is growing body of evidence that low grade inflammation plays role in developing chronic morbidities including MetS (5). Several biomarkers have been introduced to be overexpressed in inflammatory setting including C-reactive protein (CRP) (6) and cytokines (IL-6, IL-1, IL-8, IL-18) (7). Complete blood count (CBC) is one of the most accessible and inexpensive laboratory tests which provides valuable information about blood cells count and health status of patient to the physician (8). 

However, little date is available regarding any clinical significance in MetS. The present study aimed to evaluate this hypothesis in a sample of Iranian subjects enrolled in Tabari cohort study from Northern Iran. 

## Methods

The data used were extracted from the enrolment phase of Tabari cohort which is a part of nationwide Iranian cohort study, Prospective Epidemiological Research Studies in Iran (the PERSIAN Cohort Study). Detailed Questionnaires regarding demographic, medical and epidemiologic information were obtained from participants in addition to blood samples (9). The definition criteria for MetS was based on the National Cholesterol Education Program Adult Treatment Panel III criteria 3 (NCEP ATP 3) (10). To choose control subjects, those who did not have any of the items of metabolic syndrome criteria were selected. The presence of myocardial infarction, stroke, renal failure, bacterial and viral infection (HIV, hepatitis B and C), asthma, seizures, multiple sclerosis, lupus, history of cancer, and autoimmune disorders were considered the exclusion criteria. The severity score of MetS was calculated as described elsewhere (11). Contents were approved by Mazandaran University of Medical Sciences (Ethical code: IR.MAZUMS.REC.1399.836). Data analysis was carried out using SPSS software Version 20. ANOVA, chi-square, logistic regression and Pearson correlation were applied with a p<0.05. 

## Results

A total of 1930 subjects including 965 Mets positive and 965 Mets negative cases were evaluated. The distribution of MetS risk factors in case group were as follows: diabetes (818, 84.8%), hypertension (472, 48.9%), hypertriglyceridemia (788, 81.7%), low HDL cholesterol (678, 70.3%) and high waist circumference in (761, 78.9%) of patients. As presented in table 1, older age, female predominance, living in urban areas and higher body mass index (BMI) were seen in MetS cases. There were no differences between NLR, LMR and PLR in MetS and control groups (table 2). These findings were not affected by gender. In 60-70 years age category, patients had higher LMR and lower PLR however, in logistic regression analysis in table 3, no statistically association was found even after adjusting for potential confounders including age, gender, living place and BMI. Also Pearson's correlation coefficient between these ratios and MetS severity score was as follows; NLR (r =-0.069, P=0.033), LMR (r =-0.009, P=0.791) and PLR (r =-0.080, P=0.013). 

**Table 1:** Demographic data of MetS and control groups

**Item**		**MetS**	**Control**	**P-value**
**Age (Year)**	**35-39** **40-49** **50-59** **60-70**	102 (28.3%) 263 (43.5%) 343 (58.8%) 257 (67.3%)	259 (71.7%) 341 (56.5%) 240 (41.2%) 125 (32.7%)	0.00
**BMI** **(kg/m2)**	**<25** **25-29.9** **=>30**	78 (11.7%) 392 (53.3%) 495 (94.1%)	590 (88.3%) 344 (46.7%) 31 (5.9%)	0.00
**Gender (male)**		288 (34.2%)	554 (65.8%)	0.00
**Location (urban)**		713 (56.2%)	555 (43.8%)	0.00
**Smoking**		50 (24.8%)	152 (75.2%)	0.00

Body Mass Index (BMI)

**Table 2:** NLR, LMR and PLR according to age and gender in MetS and control groups

		**MetS** **Mean±SD (n)**	**Control** **Mean±SD (n)**	**P-value**
**NLR**	**Sex**			
	**Male**	1.77±0.86 (288)	1.68±0.68 (554)	0.092
	**Female**	1.61±0.63 (677)	1.69±0.78 (411)	0.062
	**Age group**			
	**35-39**	1.73±0.77	1.67±0.60	0.47
	**40-49**	1.73±0.64	1.70±0.90	0.64
	**50-59**	1.59±0.69	1.67±0.58	0.19
	**60-70**	1.65±0.77	1.71±0.64	0.41
	**Total**	1.66±0.71 (965)	1.69±0.72 (965)	0.42
**LMR**	**Sex**			
	**Male**	10.81±2.84 (288)	10.44±2.95 (554)	0.085
	**Female**	11.41±3.23 (677)	12.45±18.01 (411)	0.142
	**Age group**			
	**35-39**	12.08±3.78	11.50±3.03	0.12
	**40-49**	11.70±3.08	12.17±19.74	0.70
	**50-59**	11.06±3.19	10.56±3.19	0.06
	**60-70**	10.64±2.66	9.91±2.55	0.012
	**Total**	11.23±3.13 (965)	11.30±11.99 (965)	0.86
**PLR**	**Sex**			
	**Male**	106.02±52.50 (288)	106.84±33.50 (554)	0.782
	**Female**	117.18±74.27 (677)	123.90±36.61 (411)	0.087
	**Age group**			
	**35-39**	109.56±29.83	111.46±32.91	0.61
	**40-49**	116.15±39.57	116.04±38.48	0.97
	**50-59**	119.32±104.39	110.88±32.42	0.22
	**60-70**	105.89±33.58	120.52±39.60	0.00
	**Total**	113.85±68.67 (965)	114.11±35.85 (965)	0.91

Neutrophil-to-lymphocyte ratio (NLR), lymphocyte-to-monocyte ratio (LMR), and platelet-to-lymphocyte ratio (PLR)

**Table 3:** Logistic regression for NLR, LMR and PLR in metabolic syndrome subjects

**Ratio**	**Logistic regression P-value** **(OR, 95.0% C.I)**	
	**Univariate**	**Multivariate**
**NLR**	0.42 (0.95, 0.83-1.07)	0.15 (1.137, 0.95-1.35)
**LMR**	0.862 (0.99, 0.989-1.009)	0.782 (1.00, 0.99-1.01)
**PLR**	0.91 (1.00, 0.9-1.00)	0.814 (1.00, 0.99-1.00)

Neutrophil-to-lymphocyte ratio (NLR), lymphocyte-to-monocyte ratio (LMR), and platelet-to-lymphocyte ratio (PLR)

## Discussion

Subclinical or chronic low-grade inflammation is suspected to be a behind the scene actor in a wide range of clinical conditions (12,13). Readily available ratios calculated from CBC test have gained much attention in inflammatory settings (14). In the present report, the mean ratio values were not significantly different based on MetS positive/negative groups. The raised neutrophil count has been associated with pathologic conditions including tumor growth and metastasis (15). Heng Wan et al., in Chinese population, reported the increased prevalence of cardiovascular/cerebrovascular and kidney diseases associated with diabetes in higher NLR quartiles (16). another investigation in China failed to find any association between NLR and MetS (17). In the Turkish population, while WBC, neutrophil/lymphocyte count and hs-CRP were associated with components of MetS and its severity, NLR showed no correlation with mentioned indicators (18). Elevated monocytes and low lymphocyte count (lymphopenia) are other markers of inflammation initially studied in cancer and cardiovascular disease (19). Vahit et al., found an inverse correlation between LMR and MetS. The present investigation did not show such a result. Elevated platelet count which could be a result of IL-6 production in inflammation is a prognostic marker in malignancies (20).

 In patients with colorectal cancer, preoperative PLR was significantly higher in MetS positive patients. However, by stratifying PLR into different groups, the prevalence of MetS was not significantly different (21). One study revealed lymphocyte to high-density lipoprotein cholesterol ratio is associated with MetS even after adjusting for confounding factors, but no significant result was achieved on PLR and MetS (22). Obtained findings are in agreement with Bahadır et al. (18) and Kaya (23) and in contrast with Buyukkaya (24) and Surendar (25). Findings may be influenced by criteria for selecting MetS or control group or racial differences. As a limitation, other biologic markers of inflammation including CRP and cytokines were not available in the present study. Finally in a relatively large sample of patients with metabolic syndrome from North of Iran, no association was found between NLR, PLR and LMR with the presence of MetS. 
